# From Pressure Patterns to Personalized Insoles: A Systematic Review of Demographic Influences on Plantar Pressure

**DOI:** 10.1002/jfa2.70120

**Published:** 2026-03-31

**Authors:** Seyed Mehran Ayati Najafabadi, Hanieh Niroomand‐Oscuii, Alireza Hashemi Oskouei

**Affiliations:** ^1^ Biomedical Engineering Research Center Sahand University of Technology Tabriz Iran

**Keywords:** age, body weight, insoles, plantar pressure, sex

## Abstract

**Background:**

Plantar pressure distribution is a widely used biomechanical measure for characterizing foot–ground loading during gait in research and clinical assessment. Demographic variables, such as sex, age, and body weight, influence plantar loading patterns; however, findings across studies have been inconsistent, limiting direct clinical interpretation.

**Methods:**

A systematic review and meta‐analysis were conducted in accordance with PRISMA guidelines. PubMed, Science Direct, and Scopus were searched for studies published between 2013 and 2025 that investigated the relationship between plantar pressure and sex, age, or body weight in healthy, asymptomatic individuals. Eligible studies were screened, data were extracted, and subgroup analyses were performed to assess pressure differences across specific foot regions. Protocol registered in PROSPERO (https://www.crd.york.ac.uk/PROSPERO/view/CRD420251083389).

**Results:**

Sex‐specific differences revealed that women exhibited higher plantar pressure in the hallux, whereas men demonstrated greater loading in the heel and lateral heel regions. In older adults, a posterior‐to‐anterior shift in pressure was observed, with decreased heel loading and increased pressure in the forefoot, midfoot, and fifth metatarsal. Obesity was associated with significantly elevated plantar pressure in the first, fourth, and fifth metatarsals as well as in the heel, midfoot, and forefoot, whereas hallux pressure was relatively reduced compared to individuals of normal weight. Across all subgroup analyses, six foot regions, namely, the hallux, first, fourth, and fifth metatarsals, midfoot, and total heel consistently showed moderate to strong effect sizes.

**Conclusions:**

This study summarizes population‐specific plantar pressure patterns associated with sex, age, and body weight. The hallux, selected metatarsals, midfoot, and heel consistently demonstrated pooled differences across studies and are highlighted as regions of interest for future research. These findings provide descriptive biomechanical reference patterns that may support hypothesis‐driven investigations into plantar load redistribution and comfort‐related outcomes. These findings should therefore be interpreted as providing comparative biomechanical context rather than direct clinical decision‐making criteria.

## 1 Introduction

The human foot plays a critical role in contributing to balance, stability, and locomotion [1, 2, 3, 4, 5, 6]. Measuring plantar pressure distribution has become a reliable noninvasive method for assessing the mechanical interaction between the foot and the ground during gait. This technique is widely used in clinical and research settings to support biomechanical assessment in research and clinical settings, including characterization of loading patterns associated with pain or tissue stress [7, 8, 9, 10], as pressure‐related variables have been strongly linked to outcomes such as pain, ulceration, and tissue damage [11, 12, 13]. This study builds upon previous work by Telfer and Bigham (2019), who reviewed population characteristics and measurement systems but did not perform subgroup meta‐analyses [1].

However, interpreting plantar pressure data can be challenging due to the influence of individual factors such as sex, age, and body weight. These variables significantly alter pressure distribution patterns, often complicating clinical decision‐making and limiting the generalizability of findings. For example, although some studies report significant sex‐based differences, particularly in foot structure and plantar loading, others find no notable distinctions between men and women [2, 3, 4, 5]. Some researchers argue that women's feet are not simply scaled‐down versions of men's, highlighting structural and functional differences, particularly in arch form and pressure distribution [6].

Age‐related changes in foot anatomy and function further complicate analysis. Aging is associated with reduced joint mobility, altered foot posture, decreased plantar fascia thickness, and weakening of the ankle and intrinsic foot muscles [7]. These changes often contribute to common issues in older adults, such as foot pain, calluses, and plantar ulcers [8, 9].

Similarly, increased body weight can dramatically affect plantar loading. Obesity is often linked to a collapse of the medial longitudinal arch, resulting in flat feet and increased contact area, especially under the midfoot and forefoot [10, 11]. Obese individuals frequently report foot pain due to excessive localized pressure, an effect compounded by aging [12]. Although many studies have examined how age and body weight influence plantar pressure, their findings differ widely in terms of affected foot zones and pressure magnitude [13, 14, 15, 16].

A deeper understanding of these demographic and physiological influences is essential for both researchers and clinicians aiming to interpret plantar pressure data more accurately and design effective interventions. This review systematically evaluates how sex, age, and body weight impact plantar pressure distribution in healthy populations. In addition to identifying common high‐pressure zones associated with each factor, we aggregate results through meta‐analysis and propose reference regions of interest. Furthermore, we provide practical information on pressure measurement systems and testing protocols to support standardized assessment in both research and clinical settings.

Addressing these demographic effects is essential for advancing both evidence‐based interventions and the development of customized footwear solutions. Plantar pressure data are widely used in footwear and orthotic design to optimize load distribution and improve comfort and function. Footwear and orthotic manufacturers use plantar pressure maps to develop insoles that optimize load distribution, minimize peak pressures, and improve overall comfort and function, particularly for populations with unique biomechanical demands such as older adults, women, and individuals with obesity [17, 18]. Inadequate pressure redistribution can exacerbate existing conditions or lead to new complications, such as plantar fasciitis or metatarsalgia [19, 20]. In clinical practice, pressure mapping supports early detection of high‐risk zones in patients with diabetes or peripheral neuropathy, helping to prevent ulceration and tissue breakdown [21, 22]. However, without considering the effects of demographic variables, such as sex, age, and body weight, interventions may fail to address the underlying biomechanical factors contributing to foot pathology. Accordingly, this systematic review aims to clarify how sex, age, and body weight influence plantar pressure patterns and to propose reference regions that support the development of targeted clinical assessments and personalized footwear solutions.

## 2 Materials and Methods

This systematic review and meta‐analysis was conducted in strict adherence to the PRISMA (Preferred Reporting Items for Systematic Reviews and Meta‐Analyses) guidelines. A completed PRISMA checklist is provided in Supporting Information S1 [23]. The study protocol was preregistered in the PROSPERO database (registration number: CRD420251083389), available at https://www.crd.york.ac.uk/PROSPERO/view/CRD420251083389.

As the study exclusively involved secondary analysis of previously published data, ethical approval and informed consent were not applicable.

### 2.1 Search Strategies

A comprehensive literature search was conducted in three major electronic databases: PubMed, ScienceDirect, and Scopus. To maximize sensitivity and ensure reproducibility, the strategy combined free‐text terms with Medical Subject Headings (MeSH). In PubMed, the following Boolean syntax was applied:

((“Plantar Pressure”[MeSH] OR “plantar pressure”[All Fields] OR “foot pressure”[All Fields] OR “center of pressure”[All Fields]))

AND (“Sex Characteristics”[MeSH] OR sex [All Fields] OR gender [All Fields] OR male [All Fields] OR female [All Fields])

AND (“Aging”[MeSH] OR age [All Fields] OR elderly [All Fields] OR “older adults”[All Fields] OR “life cycle”[All Fields])

AND ((“Obesity”[MeSH] OR obesity [All Fields] OR “overweight”[All Fields] OR “body mass index”[All Fields] OR BMI [All Fields]))

The search was restricted to English‐language publications between January 2013 and August 2025, targeting studies of healthy adults aged ≥ 18 years. Both “sex” and “gender” terms were included in the search strategy; however, in the included studies, sex (biological differences) was the primary variable assessed. The complete list of keywords and search combinations is provided in Table 1.

**TABLE 1:** Search strategies for identifying relevant studies.

Row	Key words	Search strategy
1	Distribution of plantar pressure	((“Plantar Pressure”[MeSH] OR “plantar pressure”[All Fields] OR “foot pressure”[All Fields] OR “center of pressure”[All Fields]))
2	Sex	(“Sex Characteristics”[MeSH] OR sex[All Fields] OR gender[All Fields] OR male[All Fields] OR female[All Fields])
3	Age	(“Aging”[MeSH] OR age[All Fields] OR elderly[All Fields] OR “older adults”[All Fields] OR “life cycle”[All Fields])
4	Weight	(“Obesity”[MeSH] OR obesity[All Fields] OR “overweight”[All Fields] OR “body mass index”[All Fields] OR BMI [all Fields]))
Search combination		
1	Sex research	1AND2) OR 3 OR 4)
2	Age research	
3	Weight research	

**FIGURE 1:**
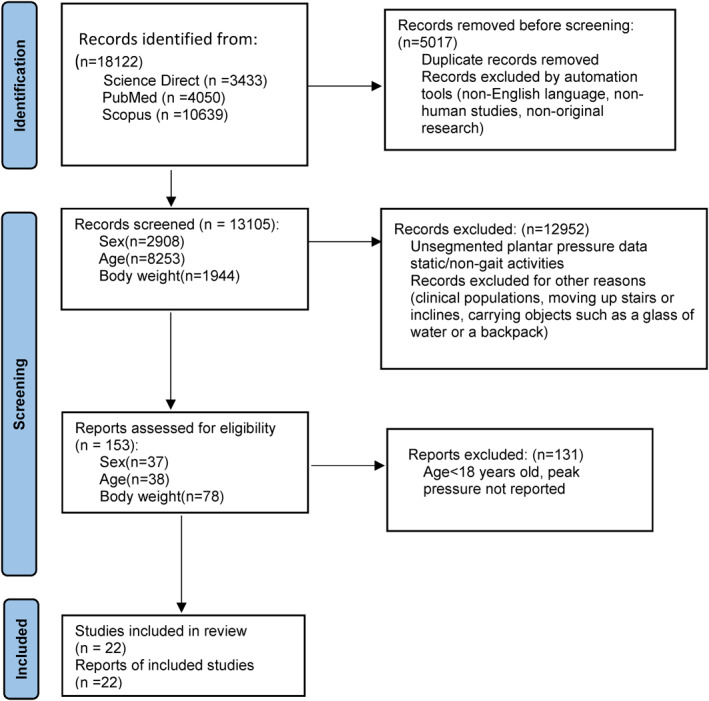
PRISMA flow diagram illustrating the study selection process, including identification, screening, eligibility, and inclusion. This process resulted in 22 studies included in the final review from 13,105 screened records.

The literature search was conducted from January 2013 to August 2025 across three databases: PubMed, Scopus, and ScienceDirect. In total, 13,105 records were initially retrieved (PubMed, *n* = 4050; Scopus, *n* = 10,639; and ScienceDirect, *n* = 3433). After removal of duplicates and application of the predefined inclusion and exclusion criteria, 22 studies were retained for the final synthesis. The PRISMA flow diagram (Figure 1) illustrates the study selection process.

Compared with Telfer and Bigham (2019) [1], our inclusion criteria were narrower, focusing on studies that provided detailed plantar regional data necessary for subgroup meta‐analyses. This methodological refinement, together with the extended search period (2013–2025), naturally resulted in a somewhat different set of included studies.

### 2.2 Inclusion and Exclusion Criteria

Eligible studies were required to meet the following criteria:

- Published in English between 2013 and 2025.
- Included healthy adults aged ≥ 18 years, as foot structure continues to mature until late adolescence [24].
- Reported plantar pressure measurements during level walking, segmented by anatomical regions.
- Examined at least one of the following variables: sex, age, or body weight.
- Eligible study designs included observational studies (cross‐sectional or cohort) conducted in healthy adults, in accordance with the PROSPERO protocol.
- Body mass index (BMI) was used as the classification variable for weight‐related analyses. Participants were categorized as normal‐weight (BMI 18.5–24.9 kg/m^2^) and obese (BMI ≥ 30 kg/m^2^). Overweight individuals (BMI 25–29.9 kg/m^2^) were excluded to enhance contrast and interpretability.

Studies were excluded if they:

- Involved clinical populations or intervention protocols (e.g., orthoses, stair use, and running).
- Lacked segmented plantar region data.
- Reported only static measurements or activities unrelated to natural gait.
- Were duplicate publications or secondary analyses of existing datasets.
- Focused primarily on clinical populations, even if they included a subgroup of healthy participants, to maintain methodological consistency and minimize bias from mixed samples.

Two reviewers independently screened titles and abstracts, with full‐text evaluations conducted where necessary. Reference lists of included studies were also searched manually for additional eligible publications. Discrepancies were resolved through discussion or, if needed, by consulting a third reviewer.

These eligibility criteria were defined a priori in the PROSPERO protocol to ensure transparency and consistency in study selection.

### 2.3 Quality of Study Selection

The methodological quality of the included studies was appraised using a biomechanically informed quality assessment tool, adapted from existing instruments [25, 26]. Specifically, the tool was designed based on general quality assessment frameworks but included additional biomechanical‐specific items, such as gait assessment protocols, plantar region segmentation, and calibration procedures. Three independent reviewers (SMA, HN, and AH) conducted the assessments. Inter‐rater disagreements were resolved through discussion and consensus. Studies that scored below 50% on the quality index were excluded from the primary meta‐analysis to ensure methodological rigor as prespecified in the PROSPERO protocol. However, these studies were retained in sensitivity analyses to test the robustness of the pooled results. The full biomechanically informed quality assessment tool, adapted from existing frameworks and expanded with gait‐specific and device‐related criteria, is provided in Supporting Information S2.

### 2.4 Data Extraction and Synthesis

Data extraction was conducted using a standardized template that included:

- Participant characteristics (age, sex, BMI, and sample size)
- Measurement systems and devices
- Reported plantar pressure values (peak pressure)
- Foot region segmentation methods

The peak plantar pressure was used as the primary outcome metric across studies, and all values were converted to kilopascals (kPa) for consistency. When numerical data were not explicitly reported but presented in figures, the graph digitization software was applied to extract values accurately.

To ensure comparability across studies, regional segmentation followed each study's original methodology but was harmonized post hoc when necessary. Specifically, plantar regions, such as the forefoot, which were reported with varying definitions across different studies, were standardized into comparable categories for meta‐analysis.

### 2.5 Meta‐Analysis Approach

The meta‐analysis was designed to quantify subgroup differences in plantar pressure by sex, age, and body weight using standardized effect size estimates.

In total, 102 data groups were extracted:

- Sex meta‐analysis: 25 groups (*n* = 3959 participants)
- Age meta‐analysis: 6 groups (*n* = 4200 participants)
- Body weight meta‐analysis: 71 groups (*n* = 6447 participants)

Only studies in which each comparison group included more than 10 participants were eligible for the meta‐regression, in accordance with Cochrane guidelines [27].

For continuous outcomes, Hedges' *g* was calculated with 95% confidence intervals. Publication bias was assessed using Egger's regression‐based test. All statistical analyses were performed using SPSS (Version 28).

Meta‐analyses were conducted using the following specifications:

- Random‐effects model for age and body weight
- Fixed‐effects model for sex
- Restricted maximum likelihood (REML) for error estimation
- Knapp–Hartung adjustment for increased precision of confidence intervals

### 2.6 Sensitivity Analysis

To evaluate the robustness of our meta‐analysis findings, we applied three approaches:

- Assessing the influence of smaller studies (*n* < 30) by temporarily excluding them and then reintroducing them in sensitivity analyses to verify the robustness of the findings.
- Excluding studies with quality scores < 50%; these studies were subsequently reintroduced in sensitivity analyses to verify the robustness of the findings.
- Performing a leave‐one‐out analysis.

Specifically, studies with fewer than 30 participants were initially excluded to assess their influence on the pooled estimates. If their removal did not meaningfully alter the direction, magnitude, or statistical significance of the effect sizes, they were reincluded in the final analysis. These complementary approaches confirmed that the overall results were stable and not overly dependent on any single study or methodological limitation.

## 3 Results

### 3.1 Study Selection

The initial screening identified 13,105 articles based on titles and abstracts. After applying the predefined inclusion and exclusion criteria, 22 studies met the eligibility requirements and were included in the final synthesis. Among these, two studies contributed data to both sex and age subgroups [28, 29], and one study contributed data to both sex and body weight subgroups [30], resulting in their inclusion across multiple analyses. The selection process is summarized in the PRISMA flow diagram (Figure 1).

### 3.2 Database

Over the past decade, a considerable number of datasets have been compiled to investigate the influence of sex, age, and body weight on plantar pressure distribution. The existing literature outlines the required conditions and experimental settings for such studies. Table 2 provides a comprehensive overview of these datasets, including research objectives, key findings, and participant demographics, whereas extended methodological details and nonsignificant results are provided in Table S1.

**TABLE 2:** Summary of studies on plantar pressure by demographic variables.

Demographic factor	Authors (ref.)	Sample size (*n*)	Significant results
Sex	[29]	700	Heel pressures were higher in men than in women across both adult (20–59 years) and older (≥ 60 years) age groups.
	[31]	20	Men exhibited higher peak pressures in the medial and lateral forefoot regions compared with women.
	[32]	100	Women showed greater peak pressures under the hallux, toes, and medial forefoot regions during walking.
	[2]	353	Peak pressures in the hallux, second metatarsal, midfoot, and both medial and lateral heel areas were higher in men than in women.
	[28]	76	Men demonstrated increased loading in the fourth and fifth metatarsals and in the medial‐lateral and lateral heel regions.
	[33]	20	Overall, male participants tended to have higher plantar pressures in posterior and lateral regions, whereas female participants exhibited higher pressures in anterior and medial areas of the foot.
Age	[29]	700	Adults exhibited lower forefoot peak pressures but higher heel pressures compared with older adults, who showed increased loading in the midfoot region.
	[34]	37	In adults, normalized peak pressure was lower in the midfoot (∼10%) than in the forefoot (∼45%) and heel (∼45%), whereas older adults displayed a distribution pattern favoring the forefoot (45%), heel (33%), and midfoot (22%).
	[28]	76	Younger adults demonstrated significantly higher pressures at the hallux, medial heel, and lateral heel, but lower pressures in the midfoot compared with older adults.
	[35]	6	Older adults showed lower pressures at the lateral heel, medial heel, fifth metatarsal, and second toe regions compared with younger participants.
	[36]	53	Peak pressures under the medial and lateral heel and second and third metatarsal regions were markedly higher in adults than in older adults.
Body weight	[13]	33	During walking, obese individuals demonstrated the highest forefoot pressures at toe‐off, whereas normal‐weight participants showed greater heel loading during initial contact.
	[37]	68	Across all plantar regions—heel, midfoot, forefoot, and hallux—obese participants exhibited higher peak pressures than normal‐weight individuals.
	[38]	34	Peak pressures under the second–fifth metatarsals and both medial and lateral heel regions were consistently greater in obese adults.
	[11]	184	Normal‐weight participants displayed relatively higher pressure beneath the hallux, whereas obese adults showed increased midfoot and heel loading.
	[39]	163	In the first metatarsal region, obese adults recorded higher pressures than normal‐weight participants, indicating greater medial foot stress.
	[40]	211	Marked differences between obese and normal‐weight groups were observed in the second‐to‐fifth toes, fourth metatarsal, midfoot, and medial heel regions.
	[15]	51	Obese volunteers showed elevated midfoot and forefoot pressures, reflecting forward load transfer during stance.
	[30]	60	Heel pressure increased progressively with rising body weight, confirming weight‐dependent loading in posterior regions.
	[16]	116	Obese participants exhibited higher pressures on both feet, particularly in the metatarsal, midfoot, and heel regions bilaterally.
	[41]	92	Overall, plantar loading patterns in obese adults showed uniformly higher pressures across anterior, central, and posterior regions compared with normal‐weight individuals.
	[42]	167	Obese participants exhibited higher bilateral pressures across both right and left feet, particularly under the first to fifth metatarsals, midfoot, and both medial and lateral heel regions.

Sample sizes among the included studies ranged from 20 to 400 participants. The majority of studies examined plantar pressure during walking (*n* = 18), whereas two assessed both standing and walking conditions [13, 32].

### 3.3 Anatomical Regions

As summarized in Table 3, more accurate assessment of plantar pressure can be achieved by subdividing the foot into smaller anatomical regions. However, there is currently no standardized approach regarding the optimal number or configuration of these regions. Variability in regional selection across studies, even when the same total number of regions was used, indicates that segmentation is often tailored to specific research objectives.

**TABLE 3:** Devices, regions measured, and statistical tests used.

Row	Name	Statistical analysis	Regions	Name and model of the device
1	[29]	Independent sample *t*‐tests	3 areas: Forefoot, midfoot, and heel	Emed ‐AT/2(Novel GmbH, Munich, Germany)
2	[31]	Student *t*‐test	5 areas: Medial and lateral forefoot, medial midfoot, and lateral heel	FDM‐TDSL (Zebris Medical, Isny Im Allgäu, Germany) treadmill
3	[32]	Two‐sample *t*‐test	10 areas: Heel, medial and lateral midfoot, hallux, second toe, fourth toe, first metatarsal, second metatarsal, third metatarsal, fourth metatarsal, and fifth metatarsal	INSOLES plantar pressure sensor (University of Fukui Graduate School of Engineering, Japan)
4	[2]	Independent samples *t*‐test	3 areas: Forefoot, midfoot, and heel	FootWork Pro system (AM Cube, France).
5	[28]	Independent sample *t*‐test Mann–Whitney *U*‐test	10 regions: Hallux, toes, first metatarsal, second metatarsal, third metatarsal, fourth metatarsal, fifth metatarsal, midfoot, medial heel, and lateral heel	(RSscan International, Olen, Belgium)
6	[33]	Two‐way ANCOVA	11 areas: Medial heel, lateral heel, medial mid, lateral mid, first metatarsal, second metatarsal, third metatarsal, fourth metatarsal, fifth metatarsal, hallux, and other fingers	emed pressure platform treadmill
1	[29]	One‐way ANOVA	3 areas: Forefoot, midfoot, and heel	Emed ‐AT/2(Novel GmbH, Munich, Germany)
2	[34]	One‐way ANOVA	3 areas: Forefoot, midfoot, and heel	mat system (Matscan, Tekscan Inc., Boston, MA, US)
3	[28]	Independent sample *t*‐test Mann–Whitney *U*‐test	10 areas: Hallux, toes, first metatarsal, second metatarsal, third metatarsal, fourth metatarsal, fifth metatarsal, midfoot, medial heel, and lateral heel	(RSscan International, Olen, Belgium)
4	[35]	ANOVA	10 areas: Hallux, second toe, third toe, fourth toe, fifth toe, first metatarsal, second metatarsal, third metatarsal, fourth metatarsal, and fifth metatarsal 6 areas: Medial and lateral heel, medial and lateral midfoot, medial and lateral forefoot	MatScan (Tekscan, Boston, MA)
5	[36]	Kruskal–Wallis test	8 areas: Hallux, other toes, first metatarsal, second to third metatarsal, fourth to fifth metatarsal, midfoot, lateral heel, and medial heel	FootWork Pro pressure plate (Am CUBE Inc., France)
1	[13]	*t*‐test	3 areas: second to third metatarsal, hallux, and heel	Tekscan Inc., US (MatScan)
2	[37]	Independent‐samples *t*‐tests	5 areas: Midfoot, forefoot, hallux, other toes, and heel	MatScan (TekScan, USA)
3	[38]	MANCOVAs	10 areas: Central and lateral and medial heel, 1st metatarsal, 3rd and 2nd metatarsal, 4th and 5th metatarsal, medial and lateral midfoot hallux, and other fingers	F‐Scan system (TekScan, Boston, MA) INSOLES
4	[11]	ANOVA	10 areas: Heel, midfoot, first metatarsal, second metatarsal, third metatarsal, fourth metatarsal, fifth metatarsal, hallux, second toe, and toes 3–5	Embed AT‐4, Novel, Germany
5	[39]	One‐way ANOVA	10 areas: Hallux, second to fifth toe, first metatarsal, second metatarsal, third metatarsal, fourth metatarsal, fifth metatarsal, midfoot, medial heel, and lateral heel	Footscan (RSscan International, Olen, Belgium)
6	[40]	One‐Way ANOVA Kruskal–Wallis test is associated with the Mann–Whitney test and Bonferroni correction.	5 areas: Heel, midfoot, forefoot, hallux, toes	Embed AT‐4 pressure platform (Novel GmbH, Munich, Germany)
7	[15]	Linear regression	5 areas: Heel, midfoot, forefoot, hallux, and toes	MatScan (Tekscan, USA)
8	[30]	Pearson correlation test (r)	4 areas: Heel, midfoot, forefoot, and toes	Emed‐x platform system (Novelgmbh 1992–2008, Germany)
9	[16]	*t*‐test Kruskal–Wallis test	2 regions: Forefoot and heel	(Tekscan Inc., MA, USA) MatScan
10	[41]	Independent sample *t*‐test Mann–Whitney *U* test(nonparametric)	10 areas: 1st toe, 2nd to 5th toe, first metatarsal, second metatarsal, third metatarsal, fourth metatarsal, fifth metatarsal, medial heel, lateral heel, and midfoot	Footscan
11	[42]	One‐way ANOVA Student–Newman–Keuls post hoc test	10 areas: 1st toe, 2nd to 5th toe, first metatarsal, second metatarsal, third metatarsal, fourth metatarsal, fifth metatarsal, medial heel, lateral heel, and midfoot	Footscan pressure plate

According to the reviewed literature, the simplest schemes divided the plantar surface into only two regions (forefoot and heel) [16], whereas the most detailed anatomical breakdown included up to 11 regions: medial and lateral heel, medial and lateral midfoot, the first through fifth metatarsals, the hallux, and a combined region for the lesser toes [33]. In some cases, extended models proposed up to 15 subdivisions, although these were sometimes collapsed or relabeled depending on study aims [35, 38].

**FIGURE 2:**
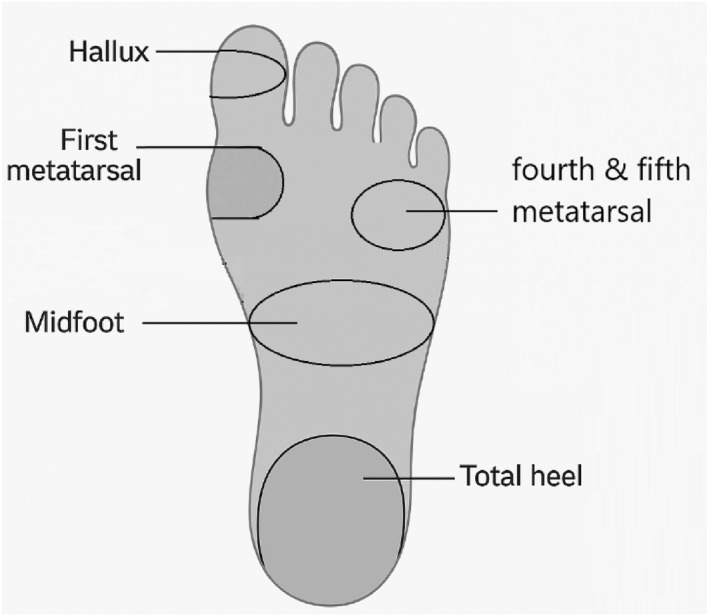
Reference plantar regions identified through pooled meta‐analysis: Hallux, first metatarsal, fourth and fifth metatarsals, midfoot, and total heel.

In the present meta‐analysis, all reported plantar regions were initially included irrespective of study design or demographic factor. By pooling these data, the regions that consistently demonstrated the strongest and most reproducible effects hallux, first metatarsal, fourth and fifth metatarsals, midfoot, and total heel were designated as reference zones. Figure 2 illustrates these reference regions for clarity, whereas extended segmentation schemes (up to 15 regions) are described in the original sources and Figure S1.

### 3.4 Pressure Measurement Systems

Various systems have been developed to measure plantar pressure and are generally categorized into three main types: ground platform systems, treadmill‐based systems, and in‐shoe systems. Table 3 summarizes the measurement tools used across the reviewed studies. Specifically, six studies employed the Emed device [11, 14, 29, 30, 33, 40], two used the Rscan system [28], and two utilized Footwork [2, 36]. Other systems included the FDM‐TDSL platform [31], in‐shoe insoles [32, 38], Matscan [13, 15, 16, 34, 35, 37], Footscan [39, 41, 42], and the F‐Scan device [38].

### 3.5 Statistical Analysis and Experimental Protocols

Table 3 also outlines the statistical methods used to analyze plantar pressure data. Eighteen studies employed inferential statistical techniques, such as ANOVA, t‐tests, ANCOVA, linear regression, or Pearson's correlation, to compare group means [2, 11, 13, 14, 15, 16, 28, 29, 30, 31, 32, 33, 34, 35, 37, 38, 39, 40, 41, 42]. Only one study reported an effect size [14]. Five studies applied nonparametric test [16, 28, 36, 40, 41], and one study [36] did not report error values, limiting comparative analysis of mean differences.

### 3.6 Gait Analysis Protocol

Fourteen studies explicitly described the approach used to collect data from a single leg per participant [2, 11, 15, 16, 28, 31, 32, 33, 34, 36, 37, 39, 40, 41], whereas six studies lacked clear justification for leg selection when pooling data [13, 28, 29, 30, 35, 38]. In seven studies [13, 28, 29, 30, 35, 38, 42], data from both legs were analyzed as paired samples. Among these, only one study applied Pearson's correlation to assess dependence between left and right leg measures [28]. Notably, two studies excluded data from one leg entirely, potentially compromising result validity [23, 28].

All included studies adopted a mid‐gait protocol. In addition, some studies implemented variations of step‐based approaches: three‐step [33, 40], two‐step [11, 29, 35], and one‐step protocols [16]. Participants were generally instructed to walk barefoot across a pressure mat at a self‐selected speed. However, two studies [28, 32] controlled walking speed using a treadmill. In‐shoe pressure monitoring devices were employed in two studies, either placed in participants' running shoes or in standardized footwear provided by the researchers [32, 38].

### 3.7 Systematic Review Findings

To identify reference regions for the quantitative synthesis, data from all plantar subdivisions were combined. This approach allowed us to pinpoint regions consistently showing strong demographic effects across studies, providing the empirical basis for selecting the hallux, selected metatarsals, midfoot, and heel as standardized reference zones. Across the 22 included studies (Table 2), consistent demographic influences on plantar pressure were observed, though the magnitude and regions of difference varied. Sex comparisons indicated that men generally exhibited higher pressures in the heel, lateral forefoot, and lateral midfoot, reflecting greater body mass and loading. In contrast, women showed elevated pressures in the hallux and forefoot. Age‐related analyses revealed a posterior‐to‐anterior shift in plantar loading with increasing age, characterized by reduced heel pressures and increased forefoot and midfoot loading. Lastly, body weight was strongly associated with elevated pressures across multiple regions, especially in the heel, midfoot, and metatarsals, often accompanied by reduced hallux contribution in obese individuals.

Taken together, these findings highlight that plantar pressure is influenced by distinct demographic factors, each with region‐specific patterns. Men and obese individuals tend to overload the heel and metatarsals, whereas aging shifts loads forward to the forefoot and midfoot. These consistent patterns offer clinically relevant insights for the design of sex, age‐, and weight‐specific footwear and orthotic interventions.

### 3.8 Regional Meta‐Analytic Differences in Plantar Pressure

**FIGURE 3:**
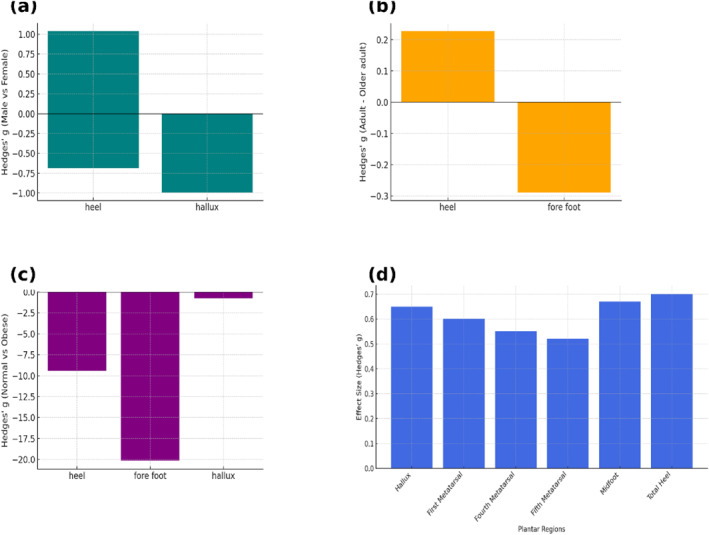
Regional meta‐analytic differences in plantar pressure distribution. (a) Sex‐related differences, showing higher heel loading in men and greater hallux pressures in women. (b) Age‐related differences, with older adults demonstrating reduced heel and increased forefoot pressures. (c) Body weight–related differences, showing higher pressures in the hallux, forefoot, and heel in obese individuals. (d) Reference plantar regions with consistent effect sizes across demographic factors (hallux, first, fourth, and fifth metatarsals, midfoot, heel).

Figure 3 summarizes the demographic influences on plantar pressure distribution. Panel (a) shows sex‐related effects, with men demonstrating higher heel loading and women exhibiting increased hallux pressures. Panel (b) highlights age‐related changes, where older adults display reduced heel loading and greater forefoot pressures relative to younger adults. Panel (c) illustrates body weight effects, with obese individuals showing markedly higher pressures in the hallux, forefoot, and heel compared with normal‐weight groups. Panel (d) presents the pooled effect sizes for reference plantar regions, identifying the hallux, first, fourth, and fifth metatarsals, midfoot, and total heel as the most consistently responsive regions.

To maintain clarity, only the most representative regions are shown in the main figure. Full regional forest plots are presented in Figures [Link], [Link], [Link], [Link] (sex, age, body weight, and reference regions), and the corresponding numerical meta‐analytic results are detailed in Tables [Link], [Link], [Link], [Link].

Full regional forest plots are presented in Figures [Link], [Link], [Link], [Link] (sex, age, body weight, and reference regions), and the corresponding numerical meta‐analytic results are detailed in Tables [Link], [Link], [Link], [Link].

### 3.9 Heterogeneity, Publication Bias, and Sensitivity Analyses

Heterogeneity varied by region and factor. Very high heterogeneity (*I* ^2^ > 90%) was observed in the hallux, midfoot, and total heel, indicating considerable methodological variability. In contrast, the lateral heel showed low heterogeneity (*I* ^2^ < 20%), reflecting stable findings. Moderate heterogeneity was seen in the first, fourth, and fifth metatarsals and the medial heel (*I* ^2^ 50%–70%), suggesting intermediate variability in demographic sensitivity.

**FIGURE 4:**
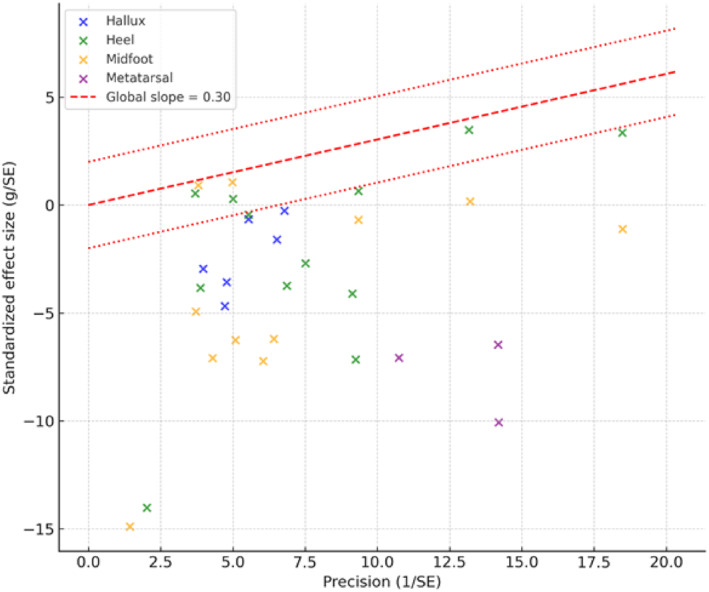
Combined Galbraith plot for hallux (blue), heel (green), midfoot (orange), and metatarsal (purple) studies with group sizes ≥ 10 per arm. Standardized effect sizes (g/SE) are plotted against precision (1/SE). The global regression line with ± 2 limits (red) shows no major evidence of publication bias across included regions.

Publication bias analyses did not raise major concerns: Egger's tests for key regions were nonsignificant, and the combined Galbraith plot demonstrated a symmetrical distribution of standardized effect sizes around the regression line with most points within ± 2 limits—indicating no major small‐study effects (Figure 4).

Sensitivity analyses confirmed robustness: excluding small‐sample comparisons (*n* < 10 per group) or low‐quality studies (< 50%) did not materially change pooled estimates; leave‐one‐out analyses similarly showed no single study unduly influenced results. Overall, subgroup differences were stable and generalizable despite methodological diversity.

## 4 Discussion

Plantar pressure distribution is a widely used biomechanical measure for examining demographic influences on foot function within research and comparative assessment contexts. The differences between our included studies and those reported by Telfer and Bigham (2019) are expected, given our updated search period (2013–2025), narrower eligibility criteria, and the requirement for detailed plantar regional data to enable subgroup meta‐analyses. These refinements extend the earlier review by providing more targeted and quantitative insights.

### 4.1 Sex Factor

Our analysis revealed sex‐specific differences in plantar pressure, notably in the hallux, total heel, and lateral heel regions. Although the hallux data exhibited heterogeneity, a moderate effect size supported distinct loading patterns between men and women in the hallux and total heel areas. The lateral heel demonstrated a pronounced and statistically significant sex difference.

These findings align with prior research indicating that men typically experience higher peak pressures, especially in the heel and lateral foot regions, compared to women [28, 29, 31, 32, 43]. This pattern may partly result from men's generally greater body mass, leading to increased mechanical loading on the plantar surface. Additionally, men tend to exhibit greater vertical displacement of the center of mass during gait, further elevating heel and lateral foot pressures [44].

Foot morphology and gait mechanics also contribute to these differences. Rislin et al. [45] reported that women display a more medial (inward) foot progression angle, whereas men show a more lateral (outward) orientation. This divergence likely contributes to higher lateral foot pressures in men and increased medial and toe region pressures in women [46]. Correspondingly, women often experience elevated forefoot and toe pressures during standing and walking, which can impact postural stability and may be linked to forefoot deformities such as hammer toes [47].

From a biomechanical perspective, these sex‐specific plantar pressure patterns may inform future research exploring footwear and orthotic design considerations. For example, differences in heel and lateral loading in men and forefoot and hallux loading in women highlight regions that may warrant further investigation in hypothesis‐driven design studies. However, these observations should be interpreted as descriptive biomechanical insights rather than direct clinical recommendations.

### 4.2 Age Factor

The study identified notable age‐related shifts in plantar pressure distribution across multiple foot regions, including the fifth metatarsal, forefoot, midfoot, and heel. Moderate effects were observed in the fifth metatarsal, forefoot, and midfoot when comparing younger and older adults, whereas medial and lateral heel regions showed strong significant differences.

Consistent with existing literature, older adults exhibit increased peak pressures in the forefoot and fifth metatarsal regions [14, 28, 29, 34], which may contribute to the higher prevalence of lower limb pain in this population [48]. Aging is associated with reduced ankle range of motion and muscle strength, alongside increased soft tissue stiffness and plantar fascia thickening [49], all of which reduce dorsiflexion during gait and increase forefoot loading during stance [8, 9].

Furthermore, age‐related declines in plantar sensitivity, especially in the midfoot and heel, may prompt a compensatory forward pressure shift to maintain balance and propulsion during mid‐stance when the body is supported on one foot [29, 50]. The shock‐absorbing capacity of the heel and midfoot also diminishes due to tissue stiffening and fat pad degeneration, further altering pressure distribution [51, 52].

In the general population, age‐related alterations in plantar pressure distribution are more commonly associated with pain, discomfort, or reduced functional capacity rather than intrinsic pathology. Such functional consequences may indirectly influence morbidity by limiting mobility and physical activity. Pathological implications become more relevant primarily in specific subpopulations, such as individuals with diabetes or peripheral neuropathy, where altered plantar loading interacts with impaired tissue tolerance and sensory deficits.

Interestingly, literature reports decreased heel and midfoot pressures with advancing age [28, 35, 36]. Although this decline in midfoot pressure during childhood corresponds with longitudinal arch development [53, 54], as children develop, these fat pads diminish and the longitudinal arch forms, resulting in a natural reduction in midfoot pressure and contact area over time [53, 55]. In older adults, it likely reflects degenerative tissue changes rather than developmental processes [14, 34, 50]. Additionally, older men may exhibit increased lateral loading under the fourth and fifth metatarsals, suggesting a lateral weight shift [14]. Scott et al. [56] also reported reduced forces under the heel, lateral forefoot, and first toe in older adults, probably related to shorter stride lengths and altered gait mechanics.

From a biomechanical perspective, these age‐related plantar pressure adaptations highlight patterns that may be relevant for future research on gait mechanics and load redistribution in older adults. Regions, such as the forefoot, midfoot, and heel, may warrant further investigation in hypothesis‐driven studies examining age‐related changes in plantar loading. These observations should be interpreted as descriptive biomechanical insights rather than direct clinical or therapeutic recommendations.

### 4.3 Body Weight Factor

This meta‐analysis demonstrated significant differences in plantar pressure between normal‐weight and obese individuals across the hallux, metatarsals, forefoot, midfoot, and heel regions. Although the hallux exhibited moderate pressure differences, all other regions showed strong and statistically significant increases in plantar loading among obese participants.

The metatarsal region bore the greatest load in obese individuals, likely due to a prolonged midstance phase during walking that extends pressure duration in these areas [38]. Interestingly, obese individuals often showed reduced hallux pressure, which may result from weakened toe flexor muscles and a compensatory gait strategy redistributing pressure away from the hallux toward the metatarsal heads [11, 13]. Structural differences, including broader and thicker feet in obese adults, further influence these loading patterns.

Excess body weight also increases pressures in the heel and midfoot, regions prone to callus formation and plantar ulceration [16, 30, 37, 41, 57]. Chronic weight‐bearing can cause medial longitudinal arch collapse, resulting in flatter feet, larger contact areas, and elevated risks of pain and dysfunction [10].

These findings parallel observations in populations with temporary weight gain, such as pregnant women, who exhibit decreased arch height and strength alongside increased foot length and arch collapse tendencies [58].

These plantar pressure alterations highlight biomechanical patterns that may be relevant for future research examining load redistribution strategies in populations with increased body mass. From a research perspective, regions, such as the metatarsals, heel, and midfoot, may warrant particular attention in hypothesis‐driven investigations of footwear or insole design. These findings should not be interpreted as prescriptive design criteria but rather as descriptive biomechanical observations.

### 4.4 Regions Factor

A key challenge in plantar pressure research is inconsistent anatomical segmentation of the foot, complicating comparisons [59, 60, 61]. Our analysis identified the hallux, first, fourth, and fifth metatarsals, midfoot, and total heel as consistently responsive regions across demographic factors, reflecting their biomechanical roles in propulsion, shock absorption, and load transfer [62, 63, 64]. Establishing these as standardized reference regions may improve methodological consistency and clinical applicability in future studies. It should be acknowledged that these reference regions reflect the current body of evidence and may be refined as additional high‐quality data and standardized methodologies become available.

The reference plantar regions proposed in this study are intended as descriptive biomechanical benchmarks derived from pooled data rather than prescriptive clinical standards. They may help standardize reporting in future research.

Although these region‐specific patterns provide useful biomechanical context, their primary value lies in supporting methodological consistency and comparative analysis in future research. The identified regions should be interpreted as descriptive biomechanical benchmarks rather than direct clinical indicators or treatment targets. Their relevance to clinical populations requires further validation through prospective and condition‐specific studies.

Accordingly, these findings may support hypothesis‐driven research exploring plantar load redistribution strategies, including exploratory investigations of footwear or orthotic interventions, without implying direct clinical efficacy or prescriptive application.

An additional consideration is the high heterogeneity (*I* ^2^ values) observed in several subgroups, which limits the strength of pooled conclusions. This variability likely reflects differences in measurement devices, gait protocols, and plantar region segmentation across studies, and findings should therefore be interpreted with caution.

## 5 Limitations

This study has some limitations. First, the age categories (“young” and “older”) vary across studies, potentially affecting comparability. Second, device reliability and measurement error were not consistently addressed, possibly influencing reported values. Third, weight‐related analyses focused primarily on average weight groups and did not extensively consider overweight or obese subgroups, which may limit conclusions about the full spectrum of weight effects. Finally, several subgroup analyses exhibited high heterogeneity (*I* ^2^), which reduces the robustness of pooled estimates and may stem from methodological or device‐related differences. In particular, variability in device resolution and calibration across studies may have contributed to inconsistencies in reported peak pressure values, and this should be taken into account when interpreting the pooled results. Nevertheless, assessment of publication bias indicated that the forest plots and the Galbraith plot demonstrated symmetrical distributions of effect sizes, suggesting that the overall findings are unlikely to be influenced by selective reporting.

## 6 Conclusion

This meta‐analysis identified consistent and biomechanically informative variations in plantar pressure distribution associated with sex, age, and body weight. Women demonstrated increased hallux pressure, whereas men exhibited greater heel and lateral heel loading. Aging was associated with an anterior shift in plantar loading, characterized by reduced heel pressure and increased loading in the forefoot, midfoot, and fifth metatarsal. Obesity was associated with elevated pressures across the metatarsals, heel, midfoot, and forefoot, alongside relatively reduced hallux loading.

Across analyses, the hallux, first, fourth, and fifth metatarsals, midfoot, and total heel consistently demonstrated medium to strong effect sizes and are therefore highlighted as descriptive regions of interest for future research rather than prescriptive clinical standards.

Overall, the demographic variations identified in this review provide biomechanically informative reference patterns that may support hypothesis‐driven research, including exploratory studies on plantar load redistribution and comfort‐related outcomes, while avoiding direct clinical or diagnostic interpretation.

## Author Contributions

**Seyed Mehran Ayati Najafabadi:** conceptualization, methodology, data curation, formal analysis, writing – original draft. **Hanieh Niroomand‐Oscuii:** conceptualization, supervision, validation, writing – review and editing. **Alireza Hashemi Oskouei:** methodology, investigation, writing – review and editing.

## Funding

The authors have nothing to report.

## Conflicts of Interest

The authors declare no conflicts of interest.

## Supporting information


Supporting Information S1

Supporting Information S2

**Figure S1**: Comprehensive 15‐region plantar segmentation scheme.

**Figure S2**: Forest plot of plantar pressure differences by sex.

**Figure S3**: Forest plot of plantar pressure differences by age.

**Figure S4**: Forest plot of plantar pressure differences by body weight.

**Figure S5**: Forest plot of plantar pressure differences by the reference region.

**Table S1**: Detailed characteristics of included studies.

**Table S2**: Meta‐analysis results for the sex subgroup.

**Table S3**: Meta‐analysis results for the age subgroup.

**Table S4**: Meta‐analysis results for the body weight subgroup.

**Table S5**: Meta‐analysis results for the reference region subgroup.

## Data Availability

All data generated or analyzed during this study are included in this published article and its supplementary files.
