# Supplementary material for: From Pressure Patterns to Personalized Insoles: A Systematic Review of Demographic Influences on Plantar Pressure
Source: J Foot Ankle Res. 2026 Mar 31;19(2):e70120. doi: 10.1002/jfa2.70120 (PMC13291806; doi:10.1002/jfa2.70120)
Supplement: Supplementary file 1 — Supporting Information S1 [file JFA2-19-e70120-s009.docx]

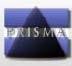
 PRISMA 2020 Checklist

| Section and Topic | Item # | Checklist item | Location where item is reported |
| --- | --- | --- | --- |
| TITLE | | |  |
| Title | 1 | From Pressure Patterns to Personalized Insoles: A Systematic Review of Demographic Influences on Plantar Pressure |  |
| ABSTRACT | | |  |
| Abstract | 2 | **Background**: Plantar pressure distribution is a widely used biomechanical measure for characterising foot–ground loading during gait in research and clinical assessment. Demographic variables such as sex, age, and body weight influence plantar loading patterns; however, findings across studies have been inconsistent, limiting direct clinical interpretation.  **Methods**: A systematic review and meta-analysis were conducted in accordance with PRISMA guidelines. PubMed, Science Direct, and Scopus were searched for studies published between 2013 and 2025 that investigated the relationship between plantar pressure and sex, age, or body weight in healthy, asymptomatic individuals. Eligible studies were screened, data were extracted, and subgroup analyses were performed to assess pressure differences across specific foot regions. Protocol registered in PROSPERO (<https://www.crd.york.ac.uk/PROSPERO/view/CRD420251083389>)  **Results**: Sex-specific differences revealed that women exhibited higher plantar pressure in the hallux, while men demonstrated greater loading in the heel and lateral heel regions. In older adults, a posterior-to-anterior shift in pressure was observed, with decreased heel loading and increased pressure in the forefoot, midfoot, and fifth metatarsal. Obesity was associated with significantly elevated plantar pressure in the first, fourth, and fifth metatarsals, as well as in the heel, midfoot, and forefoot, while hallux pressure was relatively reduced compared to individuals of normal weight. Across all subgroup analyses, six foot regions, namely the hallux, first, fourth, and fifth metatarsals, midfoot, and total heel consistently showed moderate to strong effect sizes.  **Conclusions**: This study summarises population-specific plantar pressure patterns associated with sex, age, and body weight. The hallux, selected metatarsals, midfoot, and heel consistently demonstrated pooled differences across studies and are highlighted as regions of interest for future research. These findings provide descriptive biomechanical reference patterns that may support hypothesis-driven investigations into plantar load redistribution and comfort-related outcomes. These findings should therefore be interpreted as providing comparative biomechanical context rather than direct clinical decision-making criteria. |  |
| INTRODUCTION | | |  |
| Rationale | 3 | Plantar pressure distribution is widely used in biomechanics and clinical practice but its interpretation is complicated by demographic influences (sex, age, body weight). Prior studies report inconsistent findings regarding affected regions and magnitude of loading. This inconsistency limits generalizability and clinical application, justifying a systematic review and meta-analysis. |  |
| Objectives | 4 | The objective was to synthesize current evidence on the influence of sex, age, and body weight on plantar pressure distribution in healthy individuals, to:  (i) identify consistent loading patterns,  (ii) determine foot regions most sensitive to demographic variation, and  (iii) propose descriptive reference regions to support standardized reporting and comparative biomechanical research. |  |
| METHODS | | |  |
| Eligibility criteria | 5 | Studies published in English between January 2013 and August 2025 were eligible. Participants had to be healthy adults aged ≥18 years. Studies needed to report plantar pressure distribution during normal walking, segmented by foot regions, and investigate at least one demographic factor (sex, age, or body weight/BMI). Exclusion criteria included intervention protocols (e.g., running, stair climbing, orthoses), clinical populations, duplicates, and studies with quality scores <50%. Studies were grouped for synthesis into three categories (sex, age, body weight), with overlaps included in all relevant groups. |  |
| Information sources | 6 | Electronic searches were conducted in PubMed, Scopus, and ScienceDirect as part of a single unified search covering the period from January 2013 to August 2025. Reference lists of included studies were also screened manually. |  |
| Search strategy | 7 | Search strategies combined MeSH terms and free-text keywords relating to plantar pressure, sex, age, and body weight. Searches were restricted to English-language studies of healthy adults (≥18 years) published between January 2013 and August 2025 and limited to normal walking tasks.  Data extraction: Studies were included only if they provided peak plantar pressure data, or if such data could be digitally extracted from figures. |  |
| Selection process | 8 | Titles and abstracts (n = 13,105) were screened independently by three reviewers (SMA, HN, AH). Full texts were then assessed against inclusion/exclusion criteria. Discrepancies were resolved by consensus. No automation tools were used. Twenty-four studies were finally included in the synthesis, with some contributing data to multiple subgroups. |  |
| Data collection process | 9 | Three independent reviewers extracted data on participant demographics (age, sex, BMI, sample size), measurement systems, segmentation methods, and plantar pressure values. All values were standardized to kilopascals (kPa). If data were reported only graphically, values were extracted digitally. All statistical analyses were performed in SPSS (v28). Authors of original studies were not contacted. |  |
| Data items | 10a | The main outcome was peak plantar pressure (kPa) across plantar regions. Other extracted variables included participant characteristics (age, sex, BMI), device models, and segmentation schemes. Assumptions were made only when necessary (e.g., standardizing units across studies). |  |
|  | 10b | The main outcome was peak plantar pressure (kPa) across plantar regions. Other extracted variables included participant characteristics (age, sex, BMI), device models, and segmentation schemes. Assumptions were made only when necessary (e.g., standardizing units across studies). |  |
| Study risk of bias assessment | 11 | Methodological quality was assessed using a biomechanically informed quality tool. Three reviewers independently evaluated all studies. Studies scoring <50% were excluded from the main analyses but retained in sensitivity analyses. Egger’s test was applied to assess publication bias. |  |
| Effect measures | 12 | Studies were grouped by demographic variable and plantar region. Data were standardized to kPa and extracted from text or figures. Results were summarized in tables, forest plots, funnel plots, and Galbraith plots. Random- or fixed-effects models were selected based on subgroup. Heterogeneity was assessed using I², with subgroup analysis and meta-regression to explore sources. Sensitivity analyses excluded studies with small sample sizes (n < 30) and those with low quality scores (<50%). Leave-one-out analyses were also performed to confirm robustness. |  |
| Synthesis methods | 13 a  b  c  d  e  f | Studies were grouped by demographic variable and plantar region. Data were standardized to kPa and extracted from text or figures. Results were summarized in tables, forest plots, funnel plots, and Galbraith plots. Random- or fixed-effects models were selected based on subgroup. Heterogeneity was assessed using I², with subgroup analysis and meta-regression to explore sources. Sensitivity analyses excluded studies with small sample sizes (n < 30) and those with low quality scores (<50%). Leave-one-out analyses were also performed to confirm robustness. |  |
| Reporting bias assessment | 14 | Egger’s regression test was used for publication bias. Funnel plots were generated for sex, age, weight, and reference regions. A combined Galbraith plot showed symmetrical effect size distributions, suggesting no major reporting bias. |  |
| Certainty assessment | 15 | Although a formal GRADE evaluation was not performed, methodological safeguards (exclusion of low-quality studies, consistent effect size estimation, REML and Knapp–Hartung adjustments) increase confidence in the findings. Certainty is therefore considered moderate to high, though future reviews would benefit from a formal certainty framework. |  |


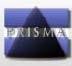
 PRISMA 2020 Checklist

| Section and Topic | Item # | Checklist item | Location where item is reported |
| --- | --- | --- | --- |
| RESULTS | |  |  |
| Study selection | 16a | After removing duplicates and screening titles and abstracts, 22 studies met the inclusion criteria and were included in the final synthesis. Several studies contributed data to multiple subgroups. | Figure 1 |
|  | 16b | Studies apparently eligible but excluded are detailed in Supplementary Table 1. Reasons for exclusion included: absence of plantar segmentation data, assessment of non-walking activities (e.g., running, stairs, orthoses), clinical populations rather than healthy adults, inability to extract peak pressure values, or methodological quality scores <50%. | Supplementary Table 1 |
| Study characteristics | 17 | The characteristics of all included studies including participant demographics, sample sizes, measurement systems, plantar region segmentation, and primary outcomes are summarized in Table 2 (main manuscript) and detailed in Supplementary Table 1. | Table2  Supplementary Table 1 |
| Risk of bias in studies | 18 | Methodological quality was assessed with a biomechanically informed quality index by three independent reviewers. Results are reported in Supplementary Table 1. Risk of publication bias was assessed with Egger’s regression test, reported in the Results and shown in Plot 1. | Plot 1  Supplementary Table 1 |
| Results of individual studies | 19 | For each outcome, individual study data including group means, standard deviations, and effect estimates with 95% confidence intervals are presented in structured tables and forest plots. Full results are available in Table 2 and Supplementary Figure 2 | Table2  Supplementary Figure 2 |
| Results of syntheses | 20a | Across studies, heterogeneity in design (segmentation schemes, devices, sample demographics) was evident. Risk of bias at the study level was moderate overall. Regions with very high heterogeneity (I² >90%) were considered lower confidence, whereas those with stable I² <20% indicated stronger reliability. |  |
|  | 20b | Sex: Women showed higher hallux pressures; men showed higher heel and lateral heel pressures. Effect sizes were moderate in hallux/total heel, and large in lateral heel.  Age: Older adults exhibited lower heel pressures but higher forefoot, midfoot, and fifth metatarsal pressures compared with younger adults.  Body weight: Obese individuals had markedly higher pressures in the heel, midfoot, forefoot, and metatarsals, with a relative reduction in hallux contribution.  Reference regions: The hallux, first, fourth, and fifth metatarsals, midfoot, and total heel consistently showed medium-to-large effects across all demographic factors. |  |
|  | 20c | Heterogeneity was explored via subgroup analysis (by demographic factor and region) and meta-regression restricted to groups with ≥10 participants. Differences in devices, segmentation schemes, and population sampling were the most plausible sources. |  |
|  | 20d | Excluding studies with small sample sizes (n<10) or quality <50% did not alter pooled estimates. Leave-one-out analyses confirmed no single study disproportionately influenced results. Subgroup findings were robust despite methodological diversity. |  |
| Reporting biases | 21 | Egger’s regression tests were non-significant across key regions. The Combined Galbraith Plot (Plot 1) for hallux (blue), heel (green), midfoot (orange), and metatarsals (purple) showed symmetrical distribution around the regression line within ±2 limits, indicating no major evidence of publication bias. | Plot 1 |
| Certainty of evidence | 22 | Formal GRADE assessment was not conducted. Certainty was higher in analyses with low heterogeneity (e.g., lateral heel in sex subgroup) and lower in regions with I² >90% (e.g., hallux, midfoot, total heel). Overall certainty was judged as moderate due to consistency of large effects but substantial heterogeneity in several subgroups. |  |
| DISCUSSION | |  |  |
| Discussion | 23a | This meta-analysis demonstrates that plantar pressure distribution is systematically influenced by demographic factors.  Sex: Women show higher pressures in the hallux, men in the heel and lateral heel.  Age: Aging shifts load anteriorly from the heel to the forefoot, midfoot, and fifth metatarsal.  Body weight: Obesity markedly increases plantar pressures in metatarsals, heel, forefoot, and midfoot, with relative hallux unloading.  Reference regions: The hallux, first, fourth, and fifth metatarsals, midfoot, and total heel consistently emerged as sensitive and clinically relevant.  These findings extend previous reviews by quantifying subgroup effects and providing standardized reference zones. |  |
|  | 23b | Variability in age group definitions (young vs. older adults).  Inconsistent reporting of device reliability/validity.  Under-representation of overweight categories (focus mainly on normal-weight vs. obese).  Non-standardized plantar segmentation across studies.  Limited reporting of participant diversity (ethnicity, activity levels, comorbidities).  Predominantly cross-sectional designs, limiting causal inference. |  |
|  | 23c | Reliance on reported and digitized data without contacting study authors.  No formal GRADE assessment.  Methodological variability in devices and region definitions was unavoidable.  Despite extended search to Aug 2025, the possibility of missing unpublished or null-result studies cannot be excluded. |  |
|  | 23d | Research: Highlights the need for standardized plantar segmentation, improved reporting of device reliability, expanded BMI categories, and longitudinal or interventional study designs to validate biomechanical findings.  Practice and policy implications are not directly inferred, as the findings are intended to provide descriptive biomechanical context rather than prescriptive clinical guidance. |  |
| OTHER INFORMATION | |  |  |
| Registration and protocol | 24a | The protocol guiding this systematic review has been formally registered in the PROSPERO database, ensuring both transparency and methodological rigor. (<https://www.crd.york.ac.uk/PROSPERO/view/CRD420251083389>) |  |
|  | 24b | If the protocol is needed, we will make it available in PDF format for reference and review. |  |
|  | 24c | The protocol amendment involved extending the predefined search period to August 2025, which was incorporated into a single unified search. No other substantive protocol changes were made. |  |
| Support | 25 | There is no financial support or reference |  |
| Competing  interests | 26 | The authors declare no conflict of interest relating to the material presented in this article. |  |
| Availability of data, code and other materials | 27 | All extracted data, statistical outputs (SPSS v28), and supplementary forest/funnel/Galbraith plots are provided in the article and Supplementary files. Additional analytic details are available upon request. |  |
