# Supplementary material for: From Pressure Patterns to Personalized Insoles: A Systematic Review of Demographic Influences on Plantar Pressure
Source: J Foot Ankle Res. 2026 Mar 31;19(2):e70120. doi: 10.1002/jfa2.70120 (PMC13291806; doi:10.1002/jfa2.70120)
Supplement: Supplementary file 2 — Supporting Information S2 [file JFA2-19-e70120-s004.docx]

# Supplementary File 2. Biomechanically-Informed Quality Assessment Tool

Purpose: To assess the methodological quality of included studies investigating plantar pressure distribution. The tool was adapted from general quality assessment frameworks but expanded with biomechanical-specific items.

| Domain | Item | Assessment Criteria | Score (Yes=1 / No=0) |
| --- | --- | --- | --- |
| Study Design & Reporting | 1. Clear statement of objectives/research question | Study states a specific aim or hypothesis | □ Yes □ No |
|  | 2. Participant characteristics | Age, sex, BMI, inclusion/exclusion criteria clearly described | □ Yes □ No |
|  | 3. Adequate sample size | ≥30 participants per group or justified by power calculation | □ Yes □ No |
|  | 4. Ethical approval reported | Institutional approval or informed consent noted | □ Yes □ No |
| Biomechanical Protocols | 5. Gait protocol adequately described | e.g., mid-gait, two-step, treadmill clearly reported | □ Yes □ No |
|  | 6. Walking condition standardized | e.g., barefoot vs in-shoe, surface type reported | □ Yes □ No |
|  | 7. Number of trials reported | ≥3 valid trials per condition, or justified | □ Yes □ No |
| Measurement System | 8. Device type and model reported | e.g., Emed, Rscan, Footscan | □ Yes □ No |
|  | 9. Device resolution adequate | ≥2 sensors/cm² or equivalent | □ Yes □ No |
|  | 10. Calibration procedure described | Reported and standardized | □ Yes □ No |
| Data Processing | 11. Plantar regions clearly segmented | Anatomical regions defined and reported | □ Yes □ No |
|  | 12. Peak pressure clearly reported | Units standardized (kPa) | □ Yes □ No |
|  | 13. Data harmonization addressed | Differences across studies discussed (e.g., forefoot boundaries) | □ Yes □ No |
| Statistical Analysis | 14. Statistical methods appropriate | e.g., ANOVA, regression, effect sizes with CI | □ Yes □ No |
|  | 15. Handling of missing data described | Drop-outs, exclusions explained | □ Yes □ No |
|  | 16. Multiple comparisons addressed | Adjustments or justification provided | □ Yes □ No |
| Overall Quality | 17. Quality score | Sum of 'Yes' responses / 16 × 100% | **%** |
|  | 18. Decision rule | ≥50% = included in primary meta-analysis; <50% = excluded (but considered in sensitivity analysis) | — |

## Instructions for Use

- Three independent reviewers (SMA, HN, AH) completed this checklist for each study.
- Disagreements resolved by consensus.
- Studies scoring <50% were excluded from the main meta-analysis but retained for sensitivity analysis.
