# Supplementary material for: From Pressure Patterns to Personalized Insoles: A Systematic Review of Demographic Influences on Plantar Pressure
Source: J Foot Ankle Res. 2026 Mar 31;19(2):e70120. doi: 10.1002/jfa2.70120 (PMC13291806; doi:10.1002/jfa2.70120)
Supplement: Supplementary file 3 — Figure S1: Comprehensive 15‐region plantar segmentation scheme. [file JFA2-19-e70120-s002.docx]

**Supplementary Figure 1: Comprehensive 15-region plantar segmentation scheme**

This figure presents the full set of 15 plantar regions used in some studies, including the hallux, toes 2–5, first through fifth metatarsals, medial and lateral midfoot, and medial, central, and lateral heel. This detailed segmentation highlights the variability in regional definitions across the literature and provides context for the harmonization strategy applied in the present meta-analysis.


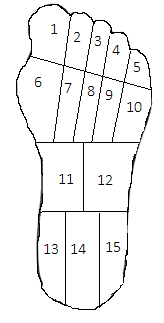


Supplementary Figure 1. Anatomical division of the plantar surface into 15 regions:

(1) Hallux, (2) Second toe, (3) Third toe, (4) Fourth toe, (5) Fifth toe, (6) First metatarsal, (7) Second metatarsal, (8) Third metatarsal, (9) Fourth metatarsal, (10) Fifth metatarsal, (11) Medial midfoot, (12) Lateral midfoot, (13) Medial heel, (14) Central heel, (15) Lateral heel.
