# Supplementary figures and images for: From Pressure Patterns to Personalized Insoles: A Systematic Review of Demographic Influences on Plantar Pressure
Source: J Foot Ankle Res. 2026 Mar 31;19(2):e70120. doi: 10.1002/jfa2.70120 (PMC13291806; doi:10.1002/jfa2.70120)

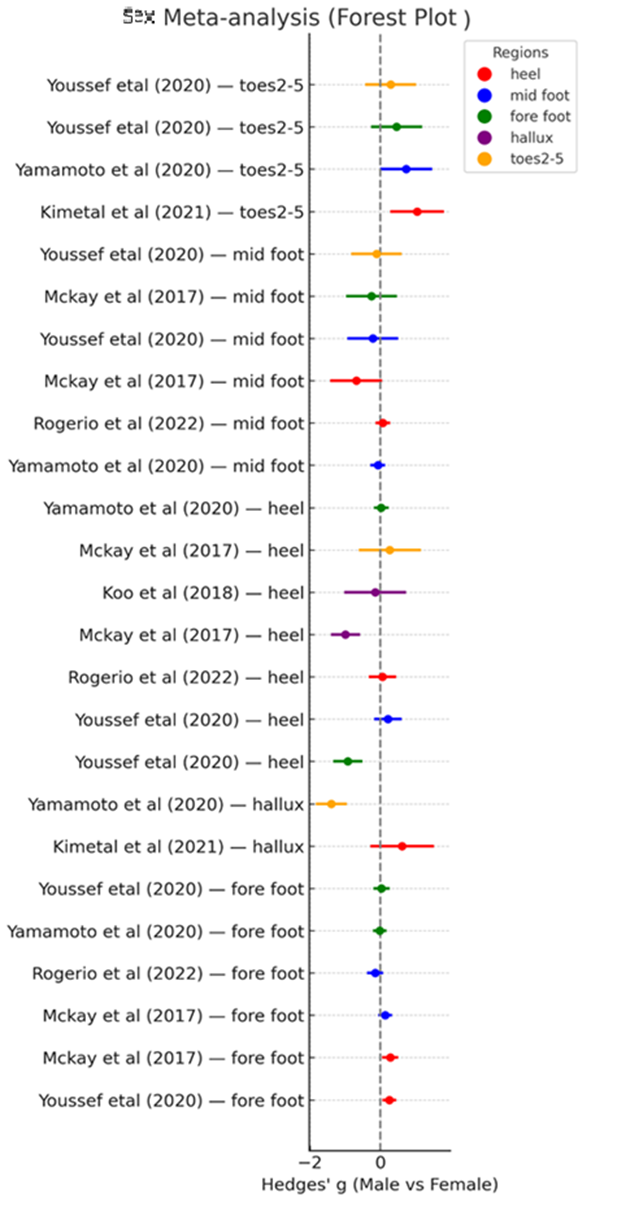

Supplement: Supplementary file 4 — Figure S2: Forest plot of plantar pressure differences by sex. [file JFA2-19-e70120-s010.docx]

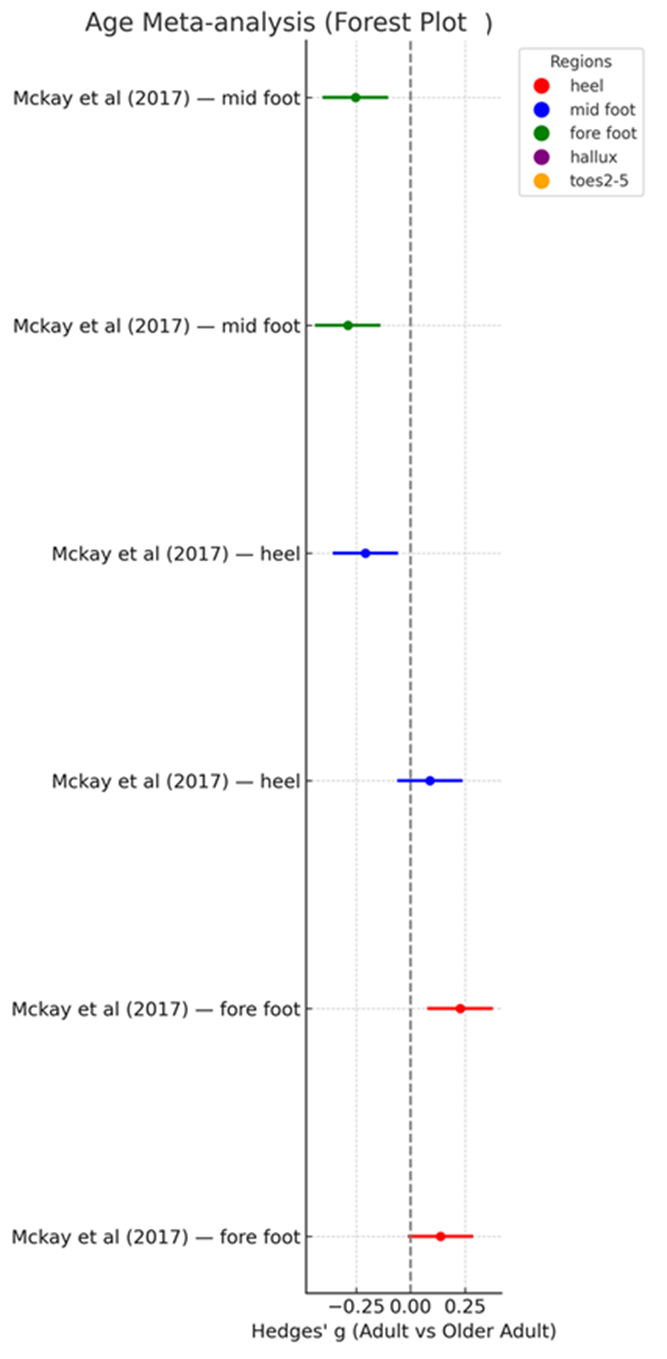

Supplement: Supplementary file 5 — Figure S3: Forest plot of plantar pressure differences by age. [file JFA2-19-e70120-s003.docx]

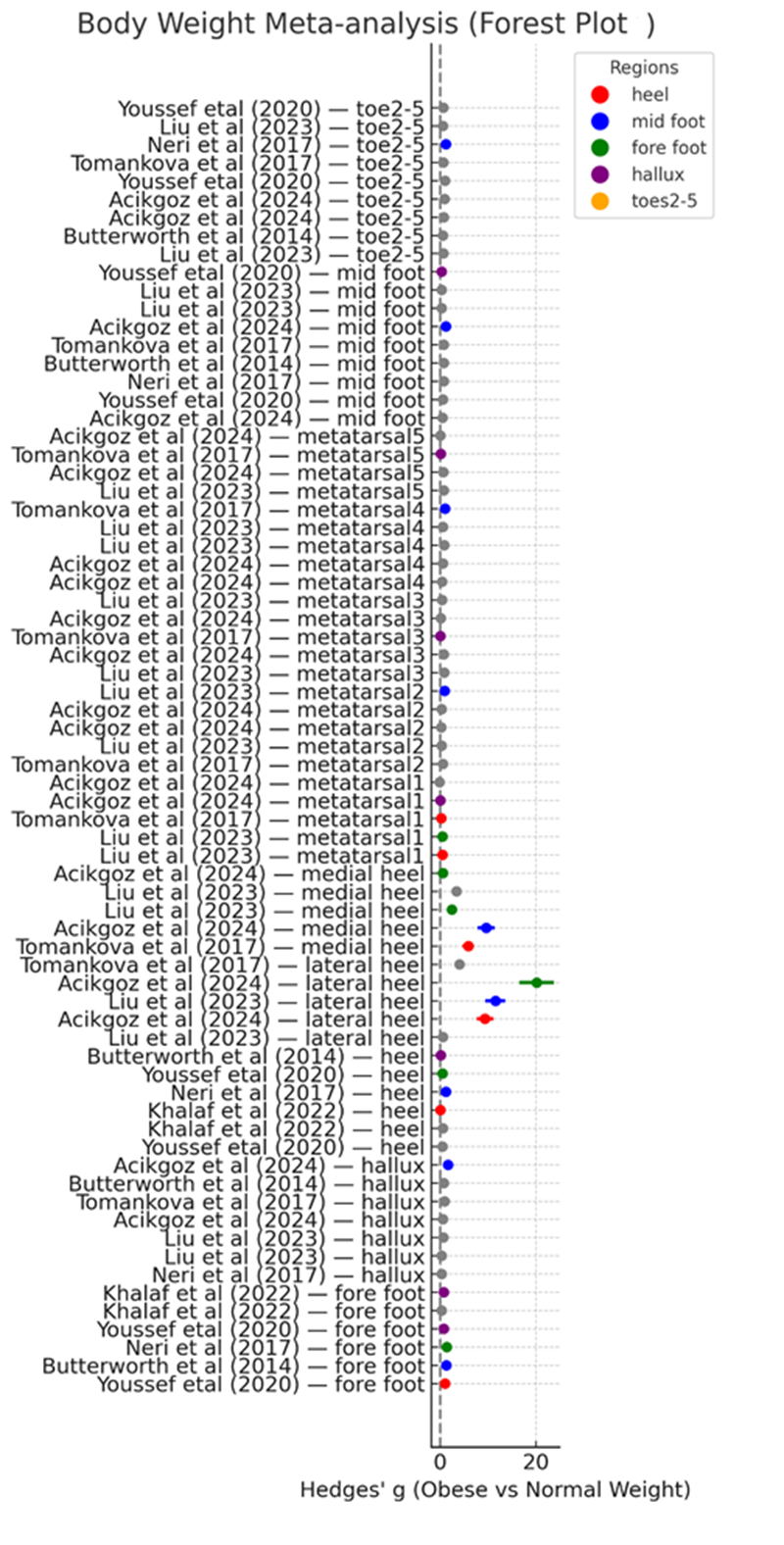

Supplement: Supplementary file 6 — Figure S4: Forest plot of plantar pressure differences by body weight. [file JFA2-19-e70120-s007.docx]

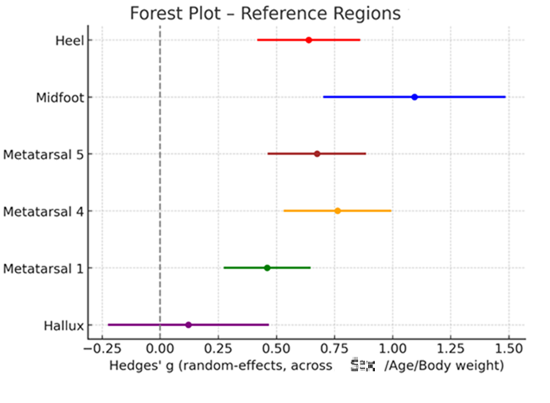

Supplement: Supplementary file 7 — Figure S5: Forest plot of plantar pressure differences by the reference region. [file JFA2-19-e70120-s006.docx]
