# Supplementary material for: From Pressure Patterns to Personalized Insoles: A Systematic Review of Demographic Influences on Plantar Pressure
Source: J Foot Ankle Res. 2026 Mar 31;19(2):e70120. doi: 10.1002/jfa2.70120 (PMC13291806; doi:10.1002/jfa2.70120)
Supplement: Supplementary file 8 — Table S1: Detailed characteristics of included studies. [file JFA2-19-e70120-s001.docx]

# Supplementary Table 1: Detailed characteristics of included studies

| Significant Results | Test Type | BMI (kg/m²) | Age (years) | Sample Size (n) | Authors (Ref.) | Row |
| --- | --- | --- | --- | --- | --- | --- |
| The pressure in the heel was higher in men than in women aged 20 to 59 and in the age group of 60 and above. | gait | BMI <40 | 3 to 101 | 70000 | [29] | 1 |
| Men had higher peak pressure in the medial and outer forefoot than women. | gait | BMI = 23.9 ± 1.8  BMI = 20.0 ± 1.9 | 22.4±1.2 | 20  (10 men, 10 women) | [37] | 2 |
| Significantly higher peak pressures were observed on the hallux, toes, forefoot, and medial aspect of the foot in women than in men during walking. | static  gait | BMI = 23.1 ± 2.7  BMI = 20.5 ± 2.1 | 33±6  32±9 | 100  (50 men, 50 women) | [31] | 3 |
| does not have | gait | BMI = 26.67± 3.68  BMI = 24.88± 4.58 | 20 to 64 | 353  (195 men,158 women) | [1] | 4 |
| The peak pressure in the areas of the thumb, other fingers, the second metatarsal, the midfoot, and the medial and lateral heel was higher in the male group than in the female group. | gait | BMI =22.05± 3.40 | Less than 30  More than50 | 76  (39 men, 37 women) | [28] | 5 |
| Men have more pressure than women in the fourth metatarsal, fifth metatarsal, medial-lateral, and lateral calcaneus regions. | gait | BMI = 23.5 ± 1.7  BMI = 21.1 ± 1.3 | 18 to 50 | 20  (10 men, 10 women) | [32] | 6 |
| Adults have lower peak pressure in the forefoot than older people; on the other hand, adults have the highest pressure in the heel of older people, and in the midfoot, more senior people show different behavior from adults. | gait | BMI <40 | 3 to 101 | 700 | [29] | 1 |
| In adults, normalized peak pressure was lower in the midfoot (10%) than in the forefoot (45%) and hindfoot (45%), while the forefoot and hindfoot showed similar values, and their values were different compared to the midfoot values. Among older people, high pressures were respectively in the front part of the foot (45%), then the back of the foot (33%), and finally the middle part of the foot (22%). | gait | - | 12  38  74 | 37 | [39] | 2 |
| The peak pressure in the young group was significantly higher than in the old group in the first toe, medial heel, and lateral heel areas and markedly less in the middle. | gait | BMI =22.05± 3.40 | 48 subjects with less than 30  28 subjects with more than 50 | 76  (39 men, 37 women) | [28] | 3 |
| In the areas of latral heel, medial heel, fifth metatarsal, and second toe pressure, we had lower values in older adults than adults. | gait | - | (65-66-72)  (33-38-39) | 6  (men) | [33] | 4 |
| The maximum pressure under the areas of the medial and lateral heel and the second and third metatarsal data showed a more significant difference in adults compared to older people. | gait | BMI = 21.48 (20.17 to 23.05)  BMI = 21.64 (20.50 to 22.99) | 11 subjects, 3 to 8  30 subjects, 20 to 40  12 subjects, 60 to 90 | 53 | [36] | 5 |
| In walking in Toeoff, the lowest pressure was among the normal and overweight groups; this is the case for the highest pressure in obese people. At the moment of heel stretch, the pressure was the highest in the group of ordinary people compared to obese people. | Static, gait | BMI = 25.865 (19–37) | 18 to 70 | 33 | [12] | 1 |
| The peak pressure in all areas in obese people was higher than in ordinary people in the heel, midfoot, forefoot, and hallux areas. | gait | BMI = 36.5 ± 5.1  BMI = 24± 2.4 | 52.6 | 68 | [38] | 2 |
| The pressure peak is higher in obese people than in ordinary people in the second and third metatarsals, fourth and fifth metatarsals, midfoot, and mid-heel. This is if the ordinary people were more in the area of the thumb than the obese people. | gait | BMI = 36.23 ± 3.54  BMI = 23.73± 1.14 | more than 18 | 34 | [34] | 3 |
| The pressure in the middle areas of the foot was normal and obese, respectively. Among both groups and in the area of the first metatarsus, obese people were more obese than ordinary people. | gait | BMI =22.8 ± 1.6  BMI = 33.8± 4.1 | 60 to 90 | 184 | [10] | 4 |
| The peak pressure in the second to fifth toes, fourth metatarsal, midfoot, first toe, fifth metatarsal, and medial heel, first metatarsal showed a more significant difference between the obese and average weight groups. | gait | BMI =22.5 ± 1.8  BMI = 34.6± 4.3 | 45 to 65 | 163  (women) | [42] | 5 |
| Obese volunteers had higher midfoot and forefoot pressure compared to regular-weight volunteers. | gait | BMI =22.9 ± 1.56  BMI = 33.3± 2.47 | 60 to 80 | 211  (women) | [35] | 6 |
| As body weight increases, the pressure on the heel increases | gait | BMI =28.9 ± 6.9  BMI =28. 2 ± 6.9 | 52.6 | 51  (18 men, 33 women) | [14] | 7 |
| Heel, midfoot, forefoot, and toe pressures differed between the standard and obese groups, favoring obese subjects with higher pressures in all areas measured. | gait | BMI =22.7± 0.84  BMI =37.4± 2.5 | 20 to 40 | 60  (30 men, 30 women) | [30] | 8 |
| The pressure in the front and back of the right leg and the front of the left leg was higher in obese people than in ordinary people. | gait | BMI =21.7± 1.76  BMI =29.3± 4.02 | 18 to 25 | 116  (54 men, 62 women) | [15] | 9 |
| The peak pressure in obese subjects was higher in the right foot than ordinary people in the first metatarsal, second metatarsal, fourth metatarsal, fifth metatarsal, midfoot, lateral, and medial heel in the right foot. Also, for the left foot, the pressure in obese people was higher than in ordinary people in the first metatarsal, second metatarsal, third metatarsal, fourth metatarsal, midfoot, medial, and lateral heel regions. | gait | BMI =21.9± 1.86  BMI =28.5± 2.95 | 60 to 75 | 92  (women) | [41] | 10 |
| does not have | gait | BMI = (15–25)  BMI = (30–35) | 15 to 70 | 167  (76 men, 91 women) | [40] | 11 |
