# Supplementary material for: From Pressure Patterns to Personalized Insoles: A Systematic Review of Demographic Influences on Plantar Pressure
Source: J Foot Ankle Res. 2026 Mar 31;19(2):e70120. doi: 10.1002/jfa2.70120 (PMC13291806; doi:10.1002/jfa2.70120)
Supplement: Supplementary file 9 — Table S2: Meta‐analysis results for the sex subgroup. [file JFA2-19-e70120-s005.docx]

| Sex mete- analysis | | | | | |
| --- | --- | --- | --- | --- | --- |
| CI_low | CI_high | Var_g | SE_g | Region | Study |
| 0.057041 | 0.449883 | 0.010043 | 0.100215 | heel | Mckay et al |
| 0.050946 | 0.504647 | 0.013396 | 0.11574 | heel | Mckay et al |
| -0.0599 | 0.331818 | 0.009986 | 0.099928 | mid foot | Mckay et al |
| -0.37679 | 0.075361 | 0.013304 | 0.115345 | mid foot | Mckay et al |
| -0.21983 | 0.171444 | 0.009963 | 0.099815 | fore foot | Mckay et al |
| -0.19611 | 0.255415 | 0.013268 | 0.115186 | fore foot | Mckay et al |
| -0.24759 | 1.478919 | 0.193985 | 0.440437 | heel | Koo et al |
| -1.83016 | -0.95964 | 0.049316 | 0.222072 | toes2-5 | Yamamoto et al |
| -1.33465 | -0.51472 | 0.043751 | 0.209167 | fore foot | Yamamoto et al |
| -0.17788 | 0.602372 | 0.039618 | 0.199044 | mid foot | Yamamoto et al |
| -0.33101 | 0.447141 | 0.039406 | 0.198509 | heel | Yamamoto et al |
| -1.40499 | -0.57891 | 0.044409 | 0.210734 | hallux | Yamamoto et al |
| -0.98836 | 0.693408 | 0.18406 | 0.429022 | hallux | Kimetal et al |
| -0.58103 | 1.106738 | 0.185375 | 0.430552 | toes2-5 | Kimetal et al |
| -0.19169 | 0.227016 | 0.011409 | 0.106812 | fore foot | Rogerio et al |
| -0.28262 | 0.136218 | 0.011416 | 0.106846 | mid foot | Rogerio et al |
| -0.13906 | 0.27976 | 0.011415 | 0.106843 | heel | Rogerio et al |
| -1.40591 | 0.032494 | 0.134644 | 0.366939 | heel | Youssef etal |
| -0.91602 | 0.481319 | 0.127067 | 0.356465 | mid foot | Youssef etal |
| -0.95751 | 0.441721 | 0.127411 | 0.356947 | fore foot | Youssef etal |
| -0.80996 | 0.583997 | 0.126451 | 0.3556 | toes2-5 | Youssef etal |
| 0.290093 | 1.785091 | 0.145448 | 0.381377 | heel | Youssef etal |
| 0.00963 | 1.45413 | 0.135789 | 0.368495 | mid foot | Youssef etal |
| -0.24917 | 1.163988 | 0.12996 | 0.360499 | fore foot | Youssef etal |
| -0.40771 | 0.993411 | 0.127755 | 0.357428 | toes2-5 | Youssef etal |
