# Supplementary material for: From Pressure Patterns to Personalized Insoles: A Systematic Review of Demographic Influences on Plantar Pressure
Source: J Foot Ankle Res. 2026 Mar 31;19(2):e70120. doi: 10.1002/jfa2.70120 (PMC13291806; doi:10.1002/jfa2.70120)
Supplement: Supplementary file 10 — Table S3: Meta‐analysis results for the age subgroup. [file JFA2-19-e70120-s011.docx]

| Age mete- analysis | | | | | |
| --- | --- | --- | --- | --- | --- |
| CI_low | CI_high | Var_g | SE_g | Region | Study |
| -0.01366 | 0.286071 | 0.005847 | 0.076463 | heel | Mckay et al |
| 0.076729 | 0.377066 | 0.00587 | 0.076617 | heel | Mckay et al |
| -0.06164 | 0.237895 | 0.005839 | 0.076413 | mid foot | Mckay et al |
| -0.35854 | -0.05835 | 0.005864 | 0.076579 | mid foot | Mckay et al |
| -0.43899 | -0.13808 | 0.005893 | 0.076765 | fore foot | Mckay et al |
| -0.40436 | -0.10379 | 0.005879 | 0.076678 | fore foot | Mckay et al |
