# Supplementary material for: From Pressure Patterns to Personalized Insoles: A Systematic Review of Demographic Influences on Plantar Pressure
Source: J Foot Ankle Res. 2026 Mar 31;19(2):e70120. doi: 10.1002/jfa2.70120 (PMC13291806; doi:10.1002/jfa2.70120)
Supplement: Supplementary file 11 — Table S4: Meta‐analysis results for the body weight subgroup. [file JFA2-19-e70120-s012.docx]

| Body weight mete- analysis | | | | | |
| --- | --- | --- | --- | --- | --- |
| CI_low | CI_high | Var_g | SE_g | Region | Study |
| -0.48565 | -1.49658 | 0.066508 | 0.257891 | heel | Butterworth et al |
| -0.79891 | -1.85226 | 0.072205 | 0.268711 | mid foot | Butterworth et al |
| -0.84997 | -1.91127 | 0.073301 | 0.270741 | fore foot | Butterworth et al |
| -0.24821 | -1.23468 | 0.063327 | 0.251649 | hallux | Butterworth et al |
| 0.150903 | -0.80997 | 0.060084 | 0.245119 | toe2-5 | Butterworth et al |
| -0.33715 | -1.15668 | 0.043708 | 0.209064 | hallux | Tomankova et al |
| 0.085136 | -0.71343 | 0.0415 | 0.203716 | toe2-5 | Tomankova et al |
| 0.068226 | -0.73085 | 0.041553 | 0.203846 | metatarsal1 | Tomankova et al |
| -0.2394 | -1.05257 | 0.043032 | 0.207441 | metatarsal2 | Tomankova et al |
| -0.20899 | -1.02036 | 0.042842 | 0.206983 | metatarsal3 | Tomankova et al |
| -0.53905 | -1.37455 | 0.045427 | 0.213136 | metatarsal4 | Tomankova et al |
| -0.36377 | -1.18518 | 0.043909 | 0.209546 | metatarsal5 | Tomankova et al |
| -1.19219 | -2.10382 | 0.054083 | 0.232557 | mid foot | Tomankova et al |
| -0.09884 | -0.90445 | 0.042235 | 0.205513 | medial heel | Tomankova et al |
| -0.18139 | -0.99121 | 0.042678 | 0.206587 | lateral heel | Tomankova et al |
| 0.272061 | -0.43517 | 0.03255 | 0.180415 | heel | Neri et al |
| -0.84372 | -1.61451 | 0.038664 | 0.196631 | mid foot | Neri et al |
| -0.12735 | -0.84464 | 0.033483 | 0.182982 | fore foot | Neri et al |
| 0.235645 | -0.4719 | 0.032579 | 0.180497 | hallux | Neri et al |
| -0.20981 | -0.93098 | 0.033845 | 0.18397 | toe2-5 | Neri et al |
| -7.63321 | -11.1416 | 0.801029 | 0.895002 | heel | Youssef etal |
| -9.40027 | -13.6462 | 1.173209 | 1.083148 | mid foot | Youssef etal |
| -16.4952 | -23.7704 | 3.444425 | 1.855916 | fore foot | Youssef etal |
| -3.12669 | -4.88088 | 0.200252 | 0.447496 | toe2-5 | Youssef etal |
| -4.69309 | -7.02052 | 0.352518 | 0.593732 | heel | Youssef etal |
| -7.8108 | -11.3927 | 0.834949 | 0.913755 | mid foot | Youssef etal |
| -1.80583 | -3.15164 | 0.117868 | 0.343319 | fore foot | Youssef etal |
| -2.61874 | -4.20459 | 0.163662 | 0.404552 | toe2-5 | Youssef etal |
| -0.21523 | -0.96262 | 0.036351 | 0.190659 | fore foot | Khalaf et al |
| -0.09153 | -0.83299 | 0.035777 | 0.189148 | heel | Khalaf et al |
| -0.07533 | -0.81612 | 0.035712 | 0.188976 | fore foot | Khalaf et al |
| 0.109567 | -0.62529 | 0.035142 | 0.187463 | heel | Khalaf et al |
| 0.383298 | -0.43411 | 0.043482 | 0.208523 | hallux | Liu et al |
| 0.573445 | -0.24531 | 0.043625 | 0.208865 | toe2-5 | Liu et al |
| -0.15946 | -0.99364 | 0.045285 | 0.212802 | metatarsal1 | Liu et al |
| 0.1082 | -0.71385 | 0.043977 | 0.209706 | metatarsal2 | Liu et al |
| 0.148361 | -0.67252 | 0.043852 | 0.209408 | metatarsal3 | Liu et al |
| 0.070697 | -0.7526 | 0.04411 | 0.210024 | metatarsal4 | Liu et al |
| -0.49266 | -1.35241 | 0.048104 | 0.219325 | mid foot | Liu et al |
| -0.41888 | -1.27199 | 0.047363 | 0.21763 | lateral heel | Liu et al |
| -0.3702 | -1.21923 | 0.046911 | 0.216589 | medial heel | Liu et al |
| 0.355766 | -0.46175 | 0.043494 | 0.208551 | hallux | Liu et al |
| 0.307304 | -0.5106 | 0.043534 | 0.208649 | toe2-5 | Liu et al |
| 0.050923 | -0.77309 | 0.044187 | 0.210207 | metatarsal1 | Liu et al |
| 0.040226 | -0.78419 | 0.04423 | 0.21031 | metatarsal2 | Liu et al |
| -0.12153 | -0.95353 | 0.045049 | 0.212247 | metatarsal3 | Liu et al |
| -0.37906 | -1.22881 | 0.046991 | 0.216774 | metatarsal4 | Liu et al |
| -0.16017 | -0.9944 | 0.045289 | 0.212813 | metatarsal5 | Liu et al |
| -0.57856 | -1.44675 | 0.049051 | 0.221476 | mid foot | Liu et al |
| -0.33425 | -1.18042 | 0.046595 | 0.21586 | lateral heel | Liu et al |
| -0.27631 | -1.11815 | 0.04612 | 0.214756 | medial heel | Liu et al |
| 0.278132 | -0.56949 | 0.046755 | 0.21623 | hallux | Acikgoz et al |
| 0.39074 | -0.45584 | 0.046641 | 0.215965 | toe2-5 | Acikgoz et al |
| -0.06062 | -0.91944 | 0.047999 | 0.219087 | metatarsal1 | Acikgoz et al |
| -0.15523 | -1.01936 | 0.048595 | 0.220442 | metatarsal2 | Acikgoz et al |
| -0.29318 | -1.16675 | 0.049662 | 0.222851 | metatarsal3 | Acikgoz et al |
| -0.31724 | -1.19266 | 0.049873 | 0.223323 | metatarsal4 | Acikgoz et al |
| -0.28213 | -1.15487 | 0.049568 | 0.222639 | metatarsal5 | Acikgoz et al |
| -0.75823 | -1.67811 | 0.055066 | 0.234662 | mid foot | Acikgoz et al |
| 0.155992 | -0.69426 | 0.047046 | 0.216902 | medial heel | Acikgoz et al |
| 0.123608 | -0.72761 | 0.047153 | 0.217148 | lateral heel | Acikgoz et al |
| 0.079137 | -0.77359 | 0.04732 | 0.217531 | hallux | Acikgoz et al |
| -0.20117 | -1.06823 | 0.048924 | 0.221187 | toe2-5 | Acikgoz et al |
| -0.13553 | -0.99848 | 0.048462 | 0.22014 | metatarsal1 | Acikgoz et al |
| -0.33021 | -1.20666 | 0.04999 | 0.223584 | metatarsal2 | Acikgoz et al |
| -0.45471 | -1.34187 | 0.05122 | 0.226318 | metatarsal3 | Acikgoz et al |
| -0.53982 | -1.43523 | 0.052176 | 0.22842 | metatarsal4 | Acikgoz et al |
| -0.19098 | -1.05737 | 0.048848 | 0.221017 | metatarsal5 | Acikgoz et al |
| -0.71681 | -1.63168 | 0.054469 | 0.233386 | mid foot | Acikgoz et al |
| -0.16553 | -1.0303 | 0.048666 | 0.220604 | medial heel | Acikgoz et al |
| -0.20801 | -1.07552 | 0.048975 | 0.221303 | lateral heel | Acikgoz et al |
