# Supplementary material for: From Pressure Patterns to Personalized Insoles: A Systematic Review of Demographic Influences on Plantar Pressure
Source: J Foot Ankle Res. 2026 Mar 31;19(2):e70120. doi: 10.1002/jfa2.70120 (PMC13291806; doi:10.1002/jfa2.70120)
Supplement: Supplementary file 12 — Table S5: Meta‐analysis results for the reference region subgroup. [file JFA2-19-e70120-s008.docx]

| Reference region mete- analysis | | | | | |
| --- | --- | --- | --- | --- | --- |
| **Region** | **k** | **g_RE** | **CI_low** | **CI_high** | **I2_%** |
| Hallux | 9 | 0.121892 | -0.22434 | 0.468128 | 81.97636 |
| Metatarsal 1 | 5 | 0.460652 | 0.273983 | 0.647321 | 0 |
| Metatarsal 4 | 5 | 0.762995 | 0.531408 | 0.994582 | 31.82589 |
| Metatarsal 5 | 4 | 0.674358 | 0.462389 | 0.886327 | 0 |
| Midfoot | 17 | 1.093811 | 0.701965 | 1.485658 | 96.08987 |
| Heel | 25 | 0.63932 | 0.417749 | 0.860891 | 90.35249 |
